# Amending the Structure of Renewable Carbon from Biorefinery Waste-Streams for Energy Storage Applications

**DOI:** 10.1038/s41598-018-25880-0

**Published:** 2018-05-29

**Authors:** Hoi Chun Ho, Monojoy Goswami, Jihua Chen, Jong K. Keum, Amit K. Naskar

**Affiliations:** 1 Carbon and Composite Group, Materials Science and Technology Division, Oak Ridge National Laboratory, Oak Ridge, TN 37831 USA; 2 The Bredesen Center for Interdisciplinary Research and Graduate Education, The University of Tennessee, Knoxville, TN 37996 USA; 3 Center for Nanophase Materials Sciences, Oak Ridge National Laboratory, Oak Ridge, TN 37831 USA; 4 Computer Science and Engineering Division, Oak Ridge National Laboratory, Oak Ridge, TN 37831 USA; 5 Neutron Scattering Division, Oak Ridge National Laboratory, Oak Ridge, TN 37831 USA

## Abstract

Biorefineries produce impure sugar waste streams that are being underutilized. By converting this waste to a profitable by-product, biorefineries could be safeguarded against low oil prices. We demonstrate controlled production of useful carbon materials from the waste concentrate via hydrothermal synthesis and carbonization. We devise a pathway to producing tunable, porous spherical carbon materials by modeling the gross structure formation and developing an understanding of the pore formation mechanism utilizing simple reaction principles. Compared to a simple hydrothermal synthesis from sugar concentrate, emulsion-based synthesis results in hollow spheres with abundant microporosity. In contrast, conventional hydrothermal synthesis produces solid beads with micro and mesoporosity. All the carbonaceous materials show promise in energy storage application. Using our reaction pathway, perfect hollow activated carbon spheres can be produced from waste sugar in liquid effluence of biomass steam pretreatment units. The renewable carbon product demonstrated a desirable surface area of 872 m^2^/g and capacitance of up to 109 F/g when made into an electric double layer supercapacitor. The capacitor exhibited nearly ideal capacitive behavior with 90.5% capacitance retention after 5000 cycles.

## Introduction

In the pursuit of a sustainable economy, both renewable energy and renewable chemical practices must be adopted. While the former can be produced from many sources, one feasible option for the combination of renewable energy and chemicals so far emanates from biorefineries^1^. However, with the current low oil price, biorefineries need improved profitability to compete with fossil fuels. This would require manufacturing of diversified products and effective utilization of byproducts for materials applications^1,2^. While lignin has been the center of attention for years as a co-product, the most overlooked byproduct is the impure sugar stream in liquid effluence from biorefinery pretreatment plants^3^.

There exists a state-of-the-art technology that utilizes biomass, pretreated by acids or alkali, to break down amorphous carbohydrates to sugars for better cellulose accessibility^4^. Sugar content in the biomass pretreatment liquid effluence can contain maximum of 50% of the initial hydrolysable carbohydrate from the biomass^5–7^. Therefore, the efficiency of biorefineries can be improved significantly if this waste-stream sugar can be captured in a simple, cost-effective way without a need for extensive purification and apply it to materials design. However, a challenge, for biorefinery coproduct generation from the waste-stream, is the low concentration of soluble carbohydrates^1^. Concentrating this liquid effluence using waste heat, which is widely available in biorefineries, is achievable and already a common practice in Kraft pulping mills^1,6^. Utilization of this untapped biomass sugar could be prioritized and one of the potential applications can be its conversion to carbon particles with tunable morphologies as a medium for renewable energy storage, such as electric double layer (EDL) supercapacitors.

Over the last decade, there has been growing interest in tailoring carbon sphere structures for different applications in renewable energy sectors. For EDL supercapacitor electrode applications, spherical carbon with a tunable porosity and controllable particle size distribution is of great interest^8–13^. The variety of structures can provide excellent performance for catalysis, adsorption, and energy storage^8–12,14,15^. Carbon spheres can be made from several methods^8–10,16–21^. One of the most inexpensive methods to date is hydrothermal carbonization (HTC). HTC is a relatively green technology and scalable to industrial production levels^9^. The HTC method is applicable to precursors with high moisture content much like the carbohydrates in pretreatment liquid effluence^22^. To better control the porosity, size, and shape of the carbon spheres, different strategies including templating and self-assembly were employed together with HTC^16^. Hard templating, which often uses silica as the template, can be one of the most straightforward ways to synthesize carbon spheres with a controllable morphology^14,23^. However, for silica hard templating, the most critical step is to obtain a template having strong interaction with the carbon precursor. The process is very tedious, and the removal of the template requires corrosive chemicals like sodium hydroxide or even hydrofluoric acid^13^, undesirable for green chemistry application. On the contrary, soft template synthesis does not require significant preparation or removal of the template^20,21^.

We propose the synthesis of carbonaceous matter in a controllable manner using soft templating, followed by HTC and subsequent high temperature carbonization of solid HTC-derivatives. Emulsion (made from oil, water, and surfactant) and water-based HTC were carried out at different time-scales to study the evolution of spherical carbon products. The two synthesis routes were then correlated with the resulting carbon morphology, porosity, and surface characteristics. Furthermore, the carbon products derived from renewable sugar were investigated as EDL electrodes for supercapacitor application. Supercapacitors store energy based on two different principles: EDL capacitance from the pure electrostatic charge accumulation at the electrode interface, and (2) the *pseudo*-capacitance based on fast and reversible redox processes at characteristic potentials^17^. Out of these two mechanisms, we synthesized and characterized EDL supercapacitors and hence we will discuss the EDL supercapacitors only in this article. Surface activation of carbon products was conducted using KOH. We performed large-scale molecular dynamics (MD) simulations to understand the evolution and characteristics of the pore structures in an emulsion-based system. While previous studies have shown the possibility of producing carbon spheres from carbohydrates and even acid or alkaline pretreated biomass-derived hydrolyzed hemicellulose using HTC, detailed understanding on the structural evolution with respect to the hydrothermal reaction media is not fully understood^3,7,11,24^. In this study, we used sugarcane-derived table sugar as a model molecule to establish the physics and the carbon formation mechanism. We then corroborated our findings using the result from laboratory-made steam-pretreated liquid effluence from woodchips. After establishing that perfectly hollow carbon spheres can be made from pretreatment liquid effluence, we explored the potential application of our model material as supercapacitor electrodes. This study exhibits a pathway to design sustainable energy storage materials from the waste stream of a future biorefinery.

## Results and Discussion

### Structure of the carbonaceous materials

**Figure 1:**
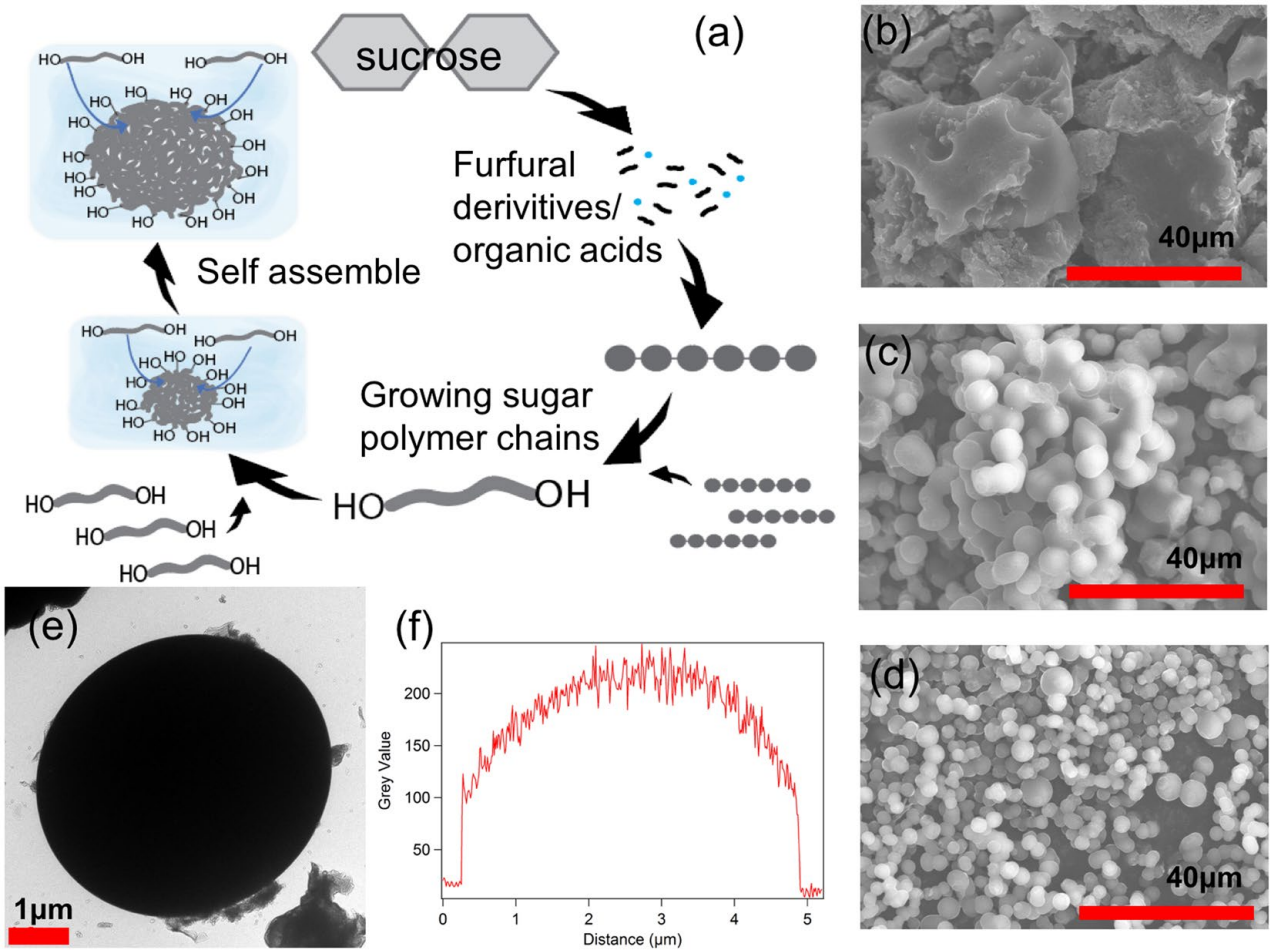
Carbon spheres from the simple HTC synthesis (N carbon samples that are made without use of any surfactant). (**a**) A schematic representation of the evolution of carbon spheres during simple HTC. SEM images of N samples with (**b**) 20, (**c**) 45, and (**d**) 165 minutes HTC durations showing carbon morphology evolving from amorphous irregular-shaped carbon to spherical particulate carbon with increasing HTC time. (**e**) TEM image of a single N carbon sphere with 45 minutes HTC duration (N45). (**f**) Thickness profile of the single N45 sphere showing a solid spherical structure formation.

The HTC of a carbohydrate precursor involves a four-step process – dehydration, condensation, polymerization, and aromatization as shown schematically in Fig. 1(a) ^23,25^. The process is as follows: sugar molecules dehydrate, forming mainly a furfural-derivative^26^ that decomposes into organic acid, and/or other species^27^. As the reaction continues, furfural and the excess dehydrated sugar condensate and polymerize. The growing “heads” in the polymer chain consist of reactive hydrophilic hydroxyl groups while the center of the chain becomes relatively dehydrated and hydrophobic. The center of the polymer chain then aromatizes with other chain centers to form a larger hydrophobic core. The aggregated chains, therefore, form spherical, micelle-like structures with a hydrophobic core and hydrophilic corona^28^. This evolution mechanism of the spherical carbonaceous aggregates was perfectly captured by scanning electron microscopy (SEM) [Fig. 1(b–d)] with our water-based HTC synthesis (abbreviated as N system representing ‘No’ surfactants). In Fig. 1, hydrothermal carbonization for 45 minutes (N45) and for 165 minutes (N165) give rise to micelle like structure and consequently spherical carbon structures [Fig. 1(c–d)]. However, the 20 minutes sample (N20) with insufficient polymerization time exhibits out-of-equilibrium amorphous irregular-shaped structures with no carbon spheres [Fig. 1(b)]. Interestingly, this shows that the micellar morphologies evolve from an irregular-shaped amorphous carbonaceous material to a perfectly shaped spherical particulate carbon as HTC time is increased. As the dehydrated sugar polymers aromatize during HTC, polymer cores continuously give off volatiles, lose functional groups, and carbonize further. Thus, when HTC duration increased, HTC samples became more carbonized and thermally stable, as seen in the thermogravimetric analysis (TGA) [Figure S1] of the HTC products. TGA results also show that carbon yield during high temperature carbonization increases as the HTC duration increases. Therefore, longer HTC time produces compact carbon spheres. For example, N45 samples show sphere diameter of 6.3 ± 1.5 μm [Fig. 1(c)] while N165 samples have spheres of diameter 3.3 ± 1.6 μm [Fig. 1(d)]. The N45 carbon, under transmission electron microscope (TEM), exhibits a perfectly spherical solid structure as shown in Fig. 1(e). The perfectly spherical structure, has been corroborated by the cross-sectional thickness profile shown in Fig. 1(f).

Ferric chloride, primarily used as a catalyst during HTC synthesis, plays a critical role in carbonization and aromatization^29–31^. When hydrolyzed, ferric chloride forms ferric hydroxide or oxide and hydrochloric acid (HCl) in water^31^. As such the resulting acid catalyzes dehydration of the sugar; reducing sugar intermediates in this system could partially reduce ferric ions to ferrous ions and then be subsequently oxidized into various iron oxide species. Therefore, it is possible that some spheres may have traces of iron oxide at the micelle core^29,31^. However, most iron was likely removed during final acid wash of the resulting carbon except any iron oxide protected within carbon shells. Inductively coupled plasma optical emission spectrometry (ICP-OES) confirms that all samples obtained after carbonization and acid washing contain <1.2% of iron with the lowest being 0.28% of Y20 [Table S1]. The low iron contents together with the minimal traces of iron redox peak in the cyclic voltammetry experiments of the supercapacitor electrodes prepared from these carbonaceous materials indicate that our energy storage device is primarily an EDL capacitor, and hence *pseudo*-capacitance plays minimal role in our results.

**Figure 2:**
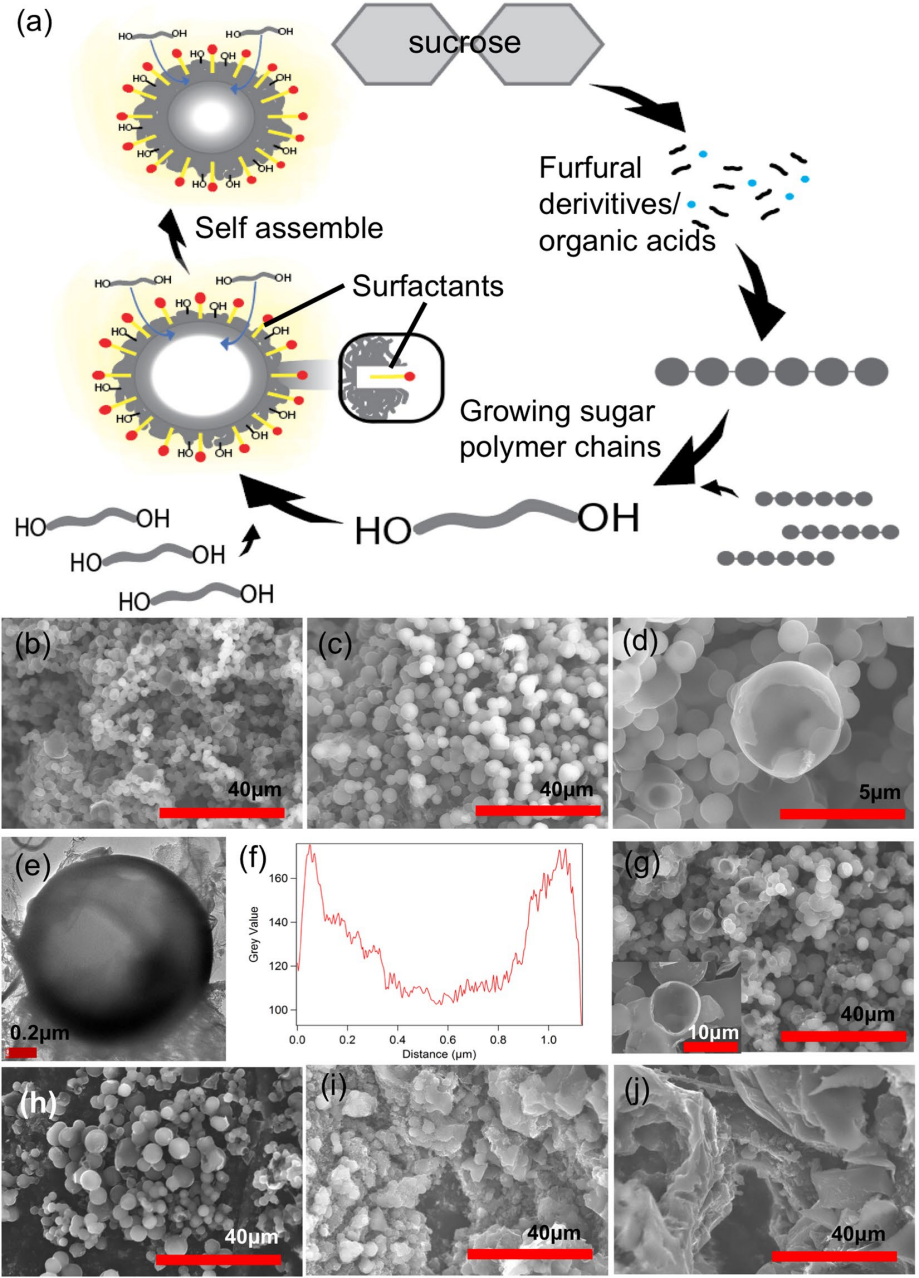
Carbon spheres from emulsion-based HTC synthesis (Y samples). (**a**) A schematic representation of the evolution of carbon spheres during emulsion-based HTC. SEM images of Y samples with (**b**) 45, (**c**) 165, and (**d**) 45 minutes HTC showing perfectly spherical structures with the longer HTC durations and a revelation of their hollow nature with broken spheres. (**e**) TEM image of a single Y sphere with 45 minutes HTC (Y45) showing its hollowness. (**f**) Thickness profile of the single Y45 sphere showing its thin shell, *ca*. 0.2 µm. SEM images of activated Y sample with (**g**) 45 minutes and (**h**) 165 minutes HTC showing the retention of carbon morphology after activation. Insert of (**g**) reveals the retention of hollow nature of spheres. (**i**) Y sample with 20 minutes HTC showing the out out-of-equilibrium structures, and (**j**) activated Y sample with 20 minutes HTC which retained the out-of-equilibrium structures after activation.

While carbon sphere formation in HTC is an established mechanism, the detailed procedure for emulsion medium HTC is far from understood. We denote emulsion synthesized carbon samples as Y samples, indicating presence of surfactant and oil in the reaction medium. The mechanism is schematically shown in Fig. 2(a). In the emulsion formed by sodium dodecyl sulfate (SDS) surfactant (1 g/100 ml), water and paraffin oil (4:1 v/v), surfactant molecules form surfactant micelles. First, sugar naturally dissolves in the water phase. As HTC progresses, the sugar molecules in water behave much like the N samples and consequently dehydrate, condensate, polymerize, and aromatize. The hydrophobicity of the dehydrated and polymerized condensed sugar molecules gradually increases. The hydrophobic polymerized sugar molecules are entropically attracted towards the hydrophobic core of the surfactant micelles in the emulsion. Note that the hydrophobic tail (dodecyl) and the hydrophilic head (sulfate) of the SDS are denoted by the yellow and red color, respectively [Fig. 2(a)]. As HTC continues, a layer-by-layer self-assembly of the sugar molecules in the surfactant micelle gives rise to the hollow carbon structures. The spherical carbon samples from emulsion-based HTC after 45 and 165 minutes (Y45 and Y165 carbons, respectively) can be seen in Fig. 2(b,c). The hollow nature of Y45 is revealed from the crumbled sphere in Fig. 2(b,d–f). The TEM image in Fig. 2(e) reveals a sphere having a bright core and dark edges indicating a hollow structure. In contrast to Fig. 1(f) where the cross-section thickness profile of N45 shows that the center of N45 sphere is the thickest part, Fig. 2(f) shows the Y45 bead having a hollow structure with the shell thickness of *ca*. 0.2 µm. Unfortunately, these broken spherical particles were not observed in the Y165 sample due to the longer HTC reaction. For long enough HTC reaction duration, the carbon shells can grow thicker and thus prevent the spheres from breaking. This mechanism also explains the smaller sizes of Y45 samples (2.5 ± 0.5 μm) as compared to 3.9 ± 1.2 μm for Y165 samples, as the longer HTC duration in Y165 allows sugar molecules to be part of a single micelle in a closely packed form. KOH activation of Y45 and Y165 [denoted as aY45 and aY165 respectively, Fig. 2(g,h)] retained their morphologies with a slight increase in size to 4.0 ± 1.7 μm and 4.14 ± 1.62 μm respectively compared to their precursors [Fig. 2(b,c)]. The slight increase in sphere sizes after activation is due to the addition of oxygen containing functional groups on carbon during activation, thereby expanding the carbon structure. Like N20, emulsion-based HTC was prematurely stopped after 20 minutes (Y20 sample) before the sugar molecules had a chance to form these hollow spherical structures, giving rise to out-of-equilibrium structures without carbon spheres [Fig. 2(i,j)]. The emulsion-based carbon bead formation will be elaborated further using Molecular Dynamics simulation in a later section.

**Figure 3:**
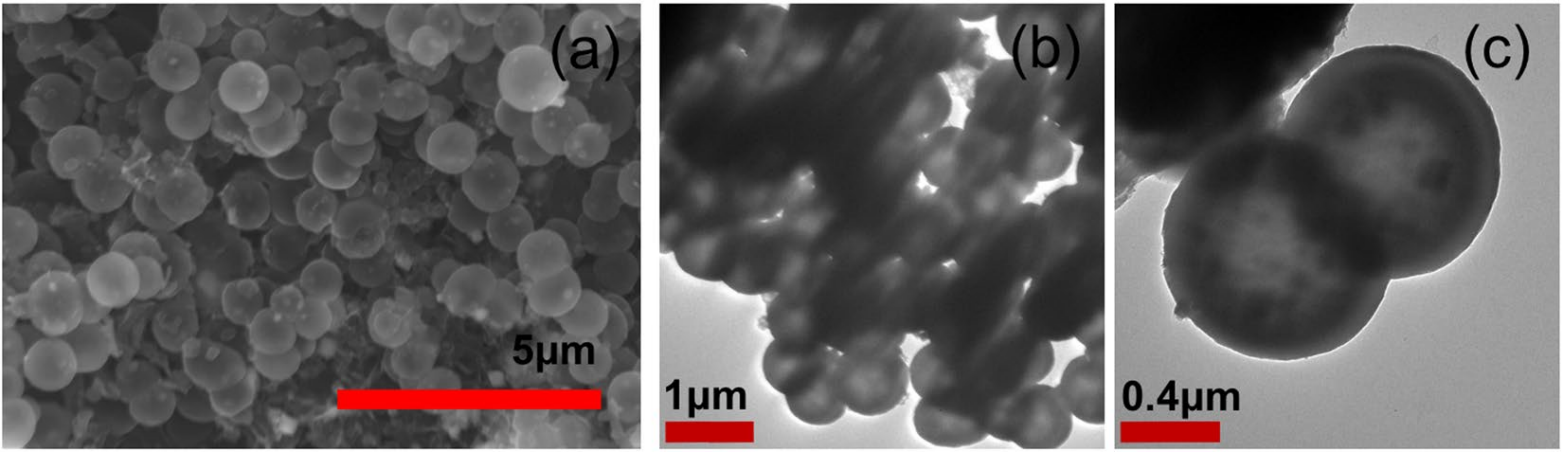
Spherical hollow carbon from steam pretreated woodchip liquid effluence. (**a**) SEM image. (**b**) and (**c**) TEM images.

So far, we discussed the pathway to produce solid and hollow carbon spheres from simple sugar molecules using water and emulsion-based HTC techniques. Subsequently, we will discuss the understanding of the self-assembly these sugar derived Y and N samples’ and energy storage properties. Prior to that, we wanted to apply the same technique to synthesize carbon from biomass pretreatment liquid effluence as our long-term goal is to utilize carbon precursors from industrial effluence to produce sustainable energy storage materials. Liquid effluence from steam pretreated woodchips was prepared and subsequently carbonized following the same emulsion medium and carbonization parameters as the Y165 sample. The resulting carbons show perfectly hollow spherical structure from the SEM and TEM images as shown in Fig. 3(a–c). These results from the biomass pretreatment liquid effluence prove that hollow carbon spheres can be produced as a co-product from biorefinery wastes, and these carbonaceous materials followed the same functionalities as that of Y samples. Carbon spheres produced herein are smaller and with thinner shells. It is known in literature that hydrothermally produced carbon sphere sizes are affected by the type of carbon precursors^32^, which can explain our smaller sphere sizes when comparing to the Y samples. For a simpler processing viewpoint, woodchip pretreatment liquid effluence was fed directly without being further concentrated into the hydrothermal synthesis reactor after being emulsified. The sugar extracted in the liquid was estimated to be ~30% of the initial woodchip mass. As a result, the sugar content in HTC used for the woodchip pretreatment effluent hydrothermal synthesis was lower than that of the Y samples, leading to the thinner carbon shell produced^11^. To the best of our knowledge, this is the first controllable hollow carbon sphere synthesis of biomass pretreatment liquid effluent from steam pretreated biomass using emulsion-based HTC. The success of this method demonstrates that our approach has potential for carbonaceous materials synthesis from a wide range of biorefinery waste materials.

### Molecular dynamics simulation

**Figure 4:**
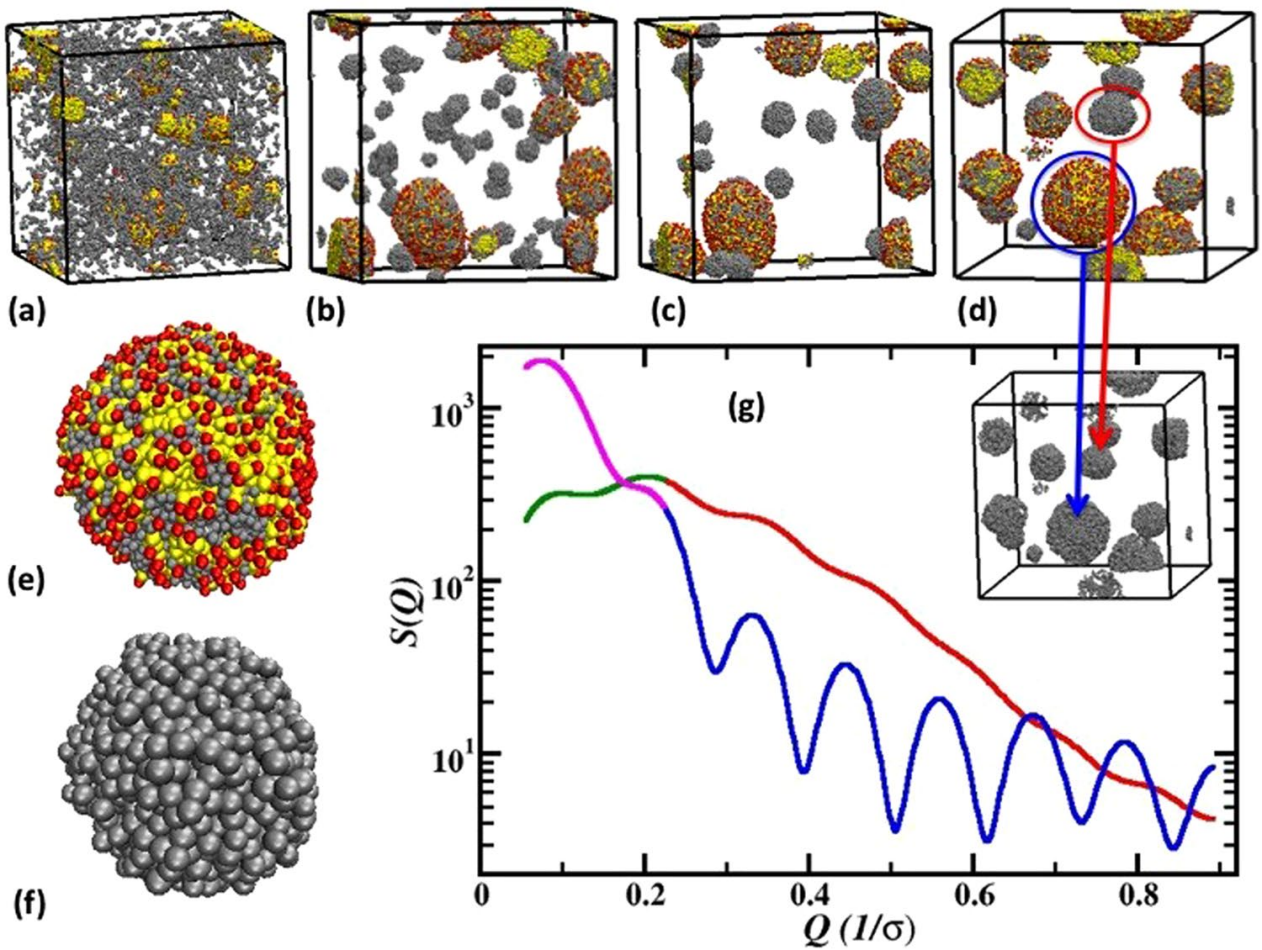
CGMD simulations of surfactant polymer mixtures replicating the surfactant-sugar emulsion HTC experiment. The top panel shows evolution of the bead formation inside the central simulation cell at (**a**) 3 million, (**b**) 4 million, (**c**) 5 million and (**d**) 8 million simulation time steps. The red and yellow color spheres represent surfactant head and tail segments. The grey color spheres represent the polymer molecules (sugar derivative in experiment). The images at (**e**) and (**f**) exhibit a single bead formed by surfactant molecules in the presence of surfactant as shown in blue circle in (d) and when the surfactants are stripped off from the same, respectively. The intermolecular structure factor, S(***Q***) for the bulk and near surfactant polymer molecules are plotted in (**g**) in red and blue color for high-*Q* range. The bulk and near surfactant polymer molecules are circled in (**d**) in red and blue colors also. The low-*Q* range profiles of S(***Q***) for the bulk and near surfactant polymers is shown in green and magenta color, respectively. The inset in (**g**) shows the snapshots for a completely stripped off surfactant system.

We believe that there is a mixture of hollow and solid spheres obtained during emulsion-based HTC, as not all Y45 and Y165 spherical carbons are formed with the assistance of surfactant micelles and therefore not all of them are hollow. To further examine the coexistence of hollow and solid spherical beads in emulsion HTC, we performed coarse-grained molecular dynamics (CGMD) simulations. The underlying principle behind the structure formation conjectured in the previous section can also be verified using computational modeling. The simulations are carried out using LAMMPS package^33^ in a canonical ensemble (see Methods Section for computational details). In an emulsion system, where surfactant number density is at or over the critical micelle number, the surfactants start forming the micelle at an early simulation stage. Figure 4(a) shows formation of such micelle at as early as 3 million simulation time steps. Figure 4(b–d) show evolution of the carbon structures at 4, 5, and a fully equilibrated 8 million simulation time steps. At the beginning of simulations (beginning of HTC in experiments), between 3–4 million time steps, it can be assumed that the charges on the polymer chains (sugar) are not fully stripped off. Therefore, when polymer (sugar) molecules are near the surfactant micelle, the micelle corona (red color beads) attracts the charges on the polymer chains. As HTC progresses, polymer chains get further dehydrated and gradually become hydrophobic. These hydrophobic backbones are then absorbed by the surfactant micelle cores due to strong interactions between many surfactant tails (which are hydrophobic) and hydrophobic polymer chains. The evolution from Fig. 4(b,c) shows a gradual change towards totally spherical beads consisting of surfactant and polymer molecules. The equilibrium structure in Fig. 4(d) consists of spherical beads formed by surfactant head and surfactant tail (red and yellow) along with the polymer chains (gray). Concurrently in Fig. 4(a–d), we also observe progress of a separate bead formation consisting of only polymer (grey color) molecules. As these polymer chains are far away from the surfactant micelle, interaction between those polymer molecules with surfactants is unfavorable. As a result, these polymer chains form individual beads with other polymer molecules only. The computer simulations show the presence of both hollow and solid spheres in an emulsion system.

The inset of Fig. 4(g) shows only the carbonaceous spherical structure after the surfactant molecules are pyrolyzed off at high temperature. Two types of spheres are observed in the emulsion-based technique, solid spheres (shown in red circle) formed by only sugar aggregated (hydrogen-bonded) beads and hollow spheres (shown in blue circle) formed by sugar polymers absorbed by surfactant micelles. Because of the difference in their formation mechanisms, we expect a difference in their morphology too. The solid spheres, away from the surfactant micelle, show smooth surfaces. Whereas, the hollow spheres are seen to exist in the surfactant micelle environment. A closer look at the single bead formed by sugar absorbed surfactant micelles reveals that the surfactants serve as templates [as in Fig. 4(e)], resulting in rough surfaces as shown in Fig. 4(f) once the surfactants are completely burnt off.

The difference in surface roughness and porosity is reiterated in detail in the structural investigation in Fig. 4(g). We show the inter-particle structure factor defined as, $\frac{1}{N}\sum _{ij}{e}^{-iQ.{r}_{ij}}$ between the polymer molecules only. Here *Q* is the wave vector, r_ij_ is the distance between two particles and N is the total number of particles. The near-surfactant S(*Q*) (blue lines), is calculated from polymer molecules within 2*σ* while the bulk S(*Q*) (red lines) is obtained from the same molecules 2*σ* distance away from the surfactant molecule, where *σ* is the Lennard-Jones diameter of each monomer. The near-surfactant S(*Q*) represents polymer molecules from only those polymer spherical beads that are agglomerated within surfactant micelles. The bulk S(*Q*) represents polymer molecules that are not associated with the surfactant micelles. As our focus is to understand the molecular level self-assembly in bulk and near-surfactant, we concentrate on S(*Q*) at high-*Q*, i.e., shorter length-scale properties. The polymer molecules in bulk show wide and weaker peaks (red), representing essentially agglomerated structures with broad distribution. The bulk molecules are parts of the sphere formed solely by polymer chains and their wider peaks represent swollen structures with a relatively smoother surface. For polymers near the surfactant molecules (blue lines), well-defined structures are observed with peak at 0.33, 0.44, 0.55, 0.66 *σ*^−1^, and so on. The structures show equally spaced molecules representing a layering of the polymer molecules within the micelle. It suggests that when the polymers enter the micelle core, they position themselves between the surfactant molecules thereby giving rise to the layering structure. As the surfactants are pyrolyzed off, the empty spaces left behind result in molecular level porous structures. This molecular level investigation corroborates our hypothesis that carbon morphology can be controlled using different formation mechanisms. The simulations concurrently support very well the collected electron micrographs and the proposed mechanism discussed in previous sections.

### Gas adsorption-desorption and surface characteristics of the carbon samples

Based on the MD simulation, we expect the rougher surface from emulsion-based Y samples to generate higher surface area due to (1) the hollow nature of the carbon spheres and (2) the templating of surfactants. We calculated the surface area and pore volumes as obtained from gas adsorption-desorption experiments in Table 1. Surface areas of Y45 and Y165 are approximately double those of N45 and N165. While no bead formation was observed with the 20 minutes HTC samples, the surfactant templating effect alone exfoliates Y20 carbons resulting in notably higher surface area than that in N20 carbons. In terms of HTC duration, surface area and pore volume both decreases as HTC duration time increases, for both Y and aY samples (Table 1) due to consolidation of layered structured pores collapsing. The trend was not as obvious with the N samples, as all three samples have surface area around 300 m^2^ g^−1^. To further increase the surface area of the carbon samples and eventually their capacitance performance, we activated Y samples with KOH. KOH acts as both the activating and templating agent, creating new pores and enlarging existing pores. As it heats up, KOH melts at 360 °C, infiltrating into macropores of carbon. As an activating agent, KOH etches new micropores and mesopores on the carbon surfaces. After washing, these emptied pores are exposed, changing the surface area and pore size distribution considerably^34^. The mechanism of KOH activation can be complex. Generally speaking, it can be represented as 6KOH + C = 2 K + 3H_2_ + 2K_2_CO_3_^35,36^. The highest surface areas and pore volumes were achieved by KOH activation. The aY20, aY45, and aY165 give rise to 1495, 1384, and 1037 m^2^g^−1^ surface areas with 0.627, 0.577, and 0.418 ccg^−1^ pore volume respectively.

**Table 1:** Surface area and pore volume measured from nitrogen adsorption isotherm.

Sample	Surface Area m^2^g^−1^	Pore Volume m^3^g^−1^	Percent micropore	Percent mesopore
aY20	1495	0.627	88.0	12.0
aY45	1384	0.577	84.1	15.9
aY165	1037	0.418	87.0	13.0
Y20	1058	0.414	91.8	8.2
Y45	743	0.347	90.1	9.9
Y165	605	0.314	81.7	18.3
N20	297	0.391	43.8	56.2
N45	273	0.416	37.7	62.3
N165	337	0.305	59.1	40.9

**Figure 5:**
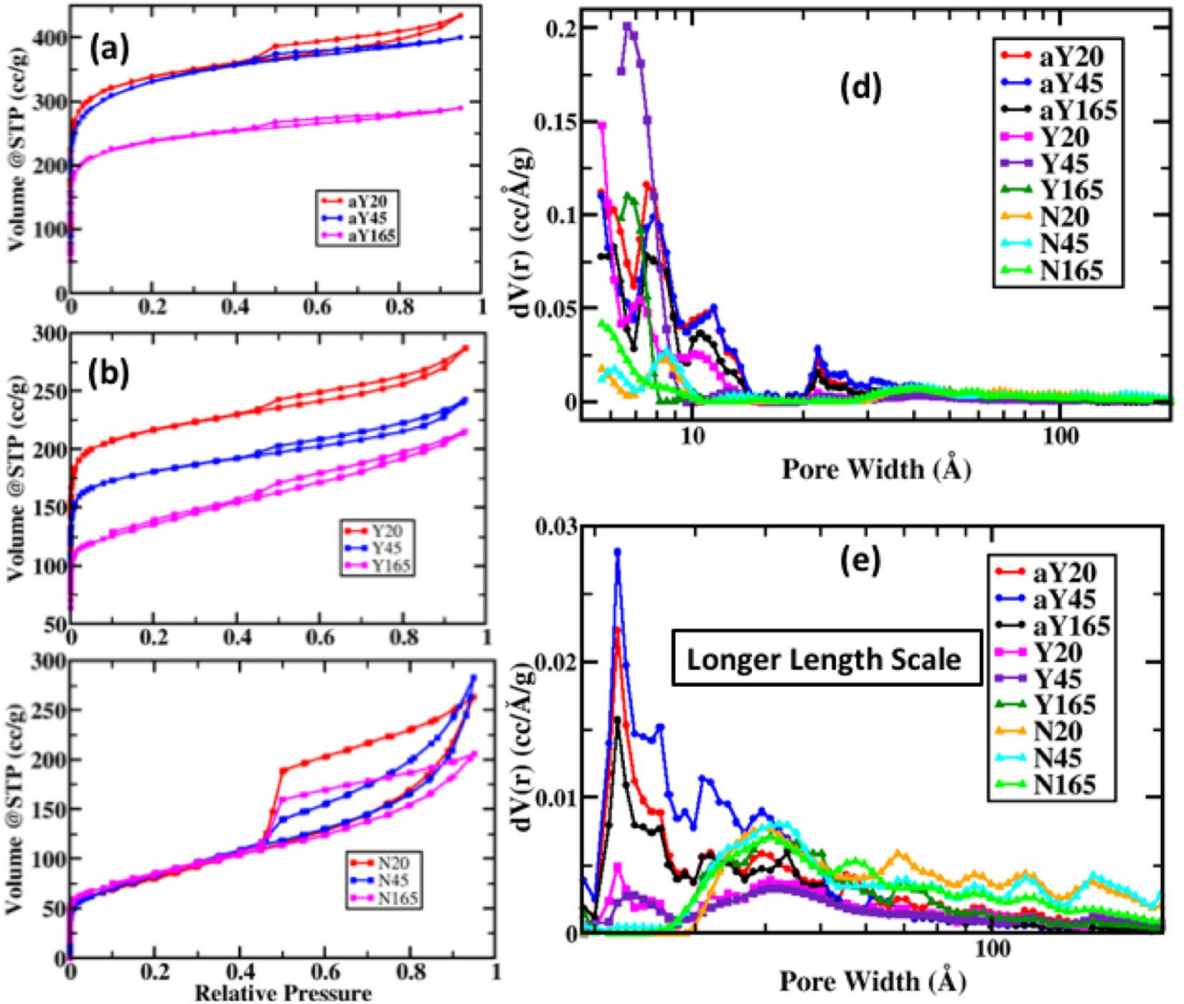
Carbon surface characteristics based on gas adsorption-desorption isotherm and porosity. (**a**), (**b**), and (**c**) Isotherms for adsorption-desorption for aY, Y and N series samples respectively. (**d**) Pore size distributions for different samples determined using the Quenched Solid Density Functional Theory (QSDFT). (**e**) Pore size distributions at longer length scales >2 nm to 200 nm. The color schemes are shown in the legends.

The molecular scale templating effect from surfactants can also be seen by the increase in microporosity which is observed in the isotherms in Fig. 5(a–c). The steep initial rise at low relative pressure suggests micropore filling^37^. Higher amounts of micropore filling (Fig. 5(a)) for the Y samples can be seen. Y and aY isotherms contain the shape of Type I isotherms while N isotherms resemble Type II isotherms^38^. The percent micropore and mesopore were also quantified in Table 1. It should be noted that the MD simulation fails to predict the mesoporosity of solid beads of the N samples. MD simulations are performed in an emulsion-based system and hence it cannot predict the N sample morphology accurately. A separate MD simulation of the N system was not performed to quantify the mesoporosity in the simulation. For Y and aY isotherms, the relatively flat plateau region suggests the limited amount of multilayer filling, or the presence of meso or macropores. N isotherms, on the contrary, have noticeable hysteresis, indicating capillary condensation in mesopores^39^. These features are also observed in the pore-size distribution analysis in Fig. 5(d) and its magnified version (from 2 nm onward) in Fig. 5(e). The mesoporous characteristics of N samples can be explained in the following: N sugar polymers were polymerized into their final shape in bulk without being templated with hydrophobic segments of a surfactant. Thus, the polymerized sugars in N samples self-assemble randomly without being fully dehydrated. During the high temperature carbonization step, these functional groups evolved as volatiles, as shown in the TGA plots in Figure S1 between *ca*. 200 °C to *ca*. 700 °C, activating and creating channels within the structure. This gives rise to the mesoporous structures in the final carbonized products. In contrast, the Y samples did not have mesopores as a result of micelle formation with the surfactant causing the sugar polymers to dehydrate before being incorporated in the hydrophobic core. This is evident by the smaller weight loss from the TGA plots in Figure S1 compared to the N counterparts between *ca*. 400 °C to *ca*. 700 °C. Hence, this mechanism suppresses the amount of mesopores that can be formed in an emulsion-based HTC as observed with the Y samples. To summarize, the layering effect between surfactants and sugar-derived carbonaceous polymers gave rise to higher surface area with smaller micropores for the Y samples while the evolution of available volatiles from activated N samples created larger mesopore channels during the high temperature carbonization step.

**Figure 6:**
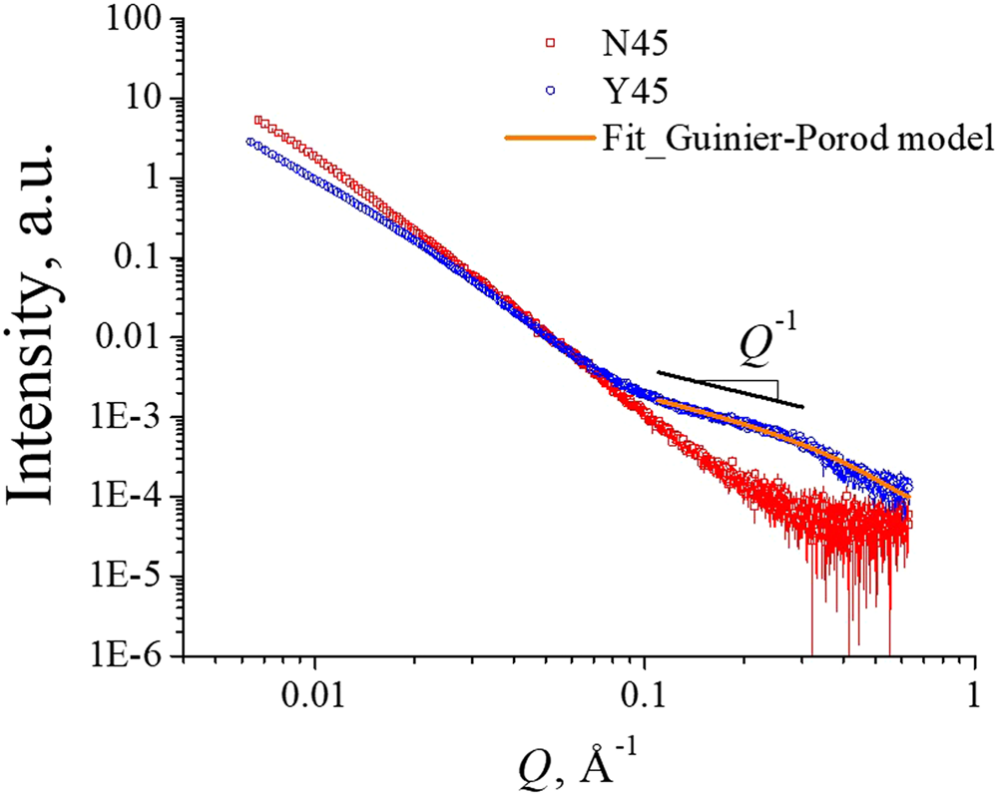
Small angle X-ray scattering (SAXS) data for N45 and Y45 carbon samples.

Small angle X-ray scattering (SAXS) characterization of two selected samples was carried out to investigate the presence of porous structure within the samples. Figure 6 depicts the SAXS curves for the carbonized N45 and Y45 samples. One of the most notable differences was that Y45 exhibits a scattering shoulder in the high-*Q* region, i.e., 0.1 < *Q* < 0.6 nm^−1^ while N45 merely shows asymptotic decay in scattering intensity. Here, *Q* is the magnitude of the scattering vector defined as *Q* = |*Q*| = 4*πλ*^−1^sin*θ*, with *λ* and *θ* being the wavelength of incident X-ray beam and half of the scattering angle, respectively. The high-*Q* scattering feature of Y45 indicates the existence of nanometer scale structures created during carbon synthesis by the assistance of the surfactant where hydrophobic carbon precursors accumulated inside the micelle and these segments were separated by the hydrophobic surfactant tail and/or the oil molecules. By considering the curve shape of the high-*Q* scattering shoulder showing Intensity ∝ *Q*^−1^, we employed the Guinier-Porod model for cylindrical objects to fit the high-*Q* scattering shoulder^40^. The data fit, indicated the existence of cylindrical pores with an average diameter of 8.6 Å (or 0.86 nm). Note that both N45 and Y45 exhibit a power law with a fractal dimension of approximately 3 in the low-Q region revealing the presence of a 3-dimensional (3D) network structure. We anticipate that they are 3D bridged pores of the samples. The N45 sample shows a steeper slope than that of the Y45 sample in low-Q region indicating larger porous structure existing within the N45 samples (see Fig. 6). These measured data corroborate very well with the measured pore volume and surface area shown in Table 1. Specifically, the pore volume and surface area of N45 and Y45 are (*ca*. 0.416 vs. 0.347 m^3^g^−1^) and (*ca*. 273 vs. 743 m^2^g^−1^), respectively.

### Supercapacitor application

To this end, we demonstrate the application of these renewable carbonaceous materials for renewable energy storage systems. We used the synthesized porous carbonaceous particles to prepare electrodes for EDL. EDL supercapacitors, are energy storage devices with power performances that fit in between dielectric capacitors and batteries from the Ragone plot, which exemplifies the energy and power relationships for different energy storage devices^41^. Unlike batteries and pseudo-capacitors, EDL supercapacitors do not rely on faradaic reactions^42^. Thus, these type of supercapacitors have higher charge-discharge rates and stabilities^43^. EDL supercapacitors rely solely on the electrostatic separation between ions in electrolyte and electrons in electrodes^44^. Using porous carbonaceous materials to serve as electrodes has gathered significant interest^45,46^, especially when derived from renewable materials^47–49^.

**Figure 7:**
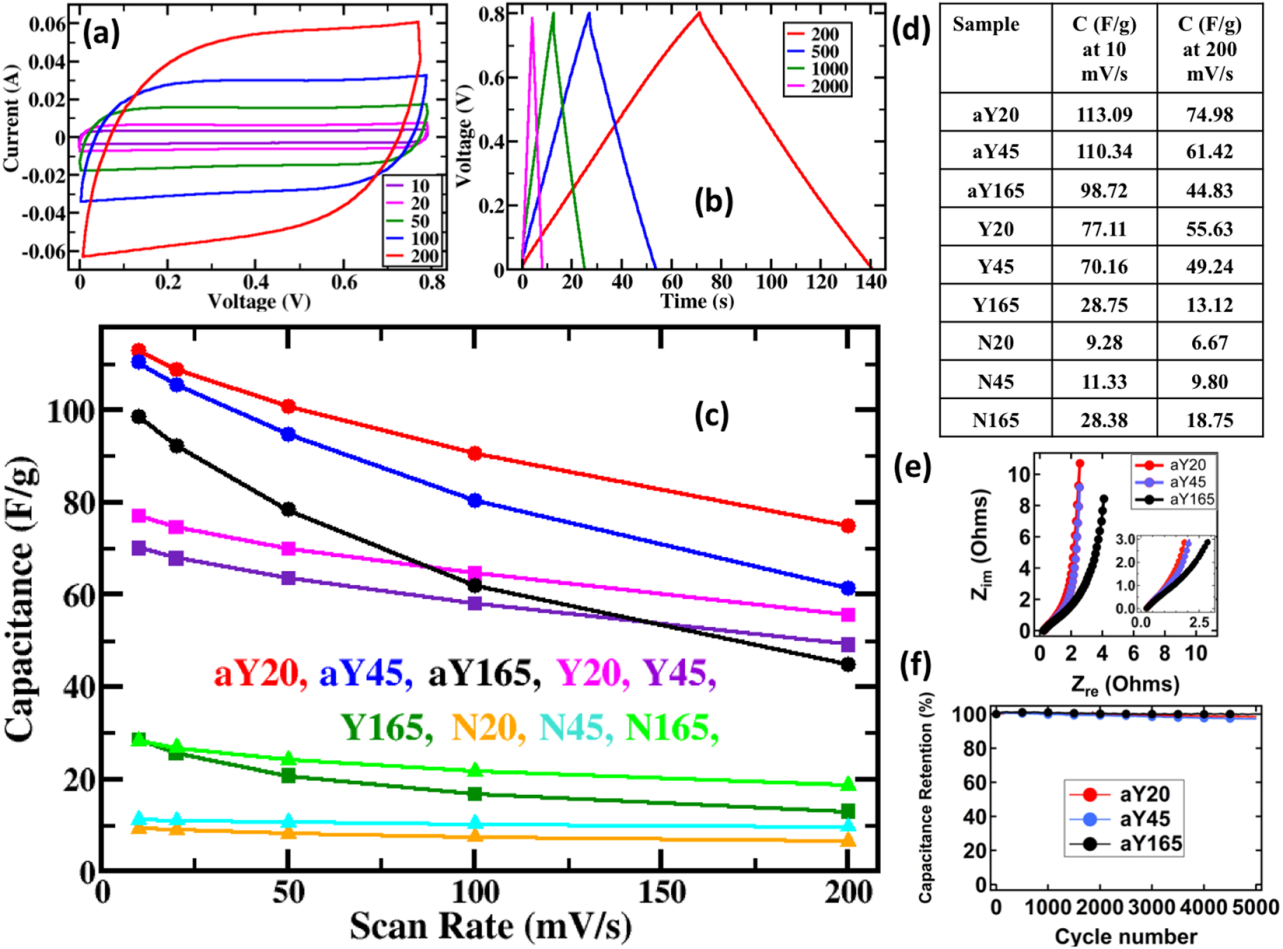
Capacitance measurement using carbonaceous materials as electrodes of EDL supercapacitors. (**a**) Cyclic voltammetry IV curves for Y45 at 10, 20, 50, 100 and 200 mVs^−1^ scan rates. The legends are in mVs^−1^ (**b**) Charge-discharge experiments for Y45 sample at 200, 500, 1000 and 2000 mAg^−1^ current densities. Legends are in mAg^−1^. (**c**) Capacitances for all the samples as shown in the legend color code. (**d**) Table showing the capacitance values at two different scan rates. (**e**) Electrochemical impedance spectroscopy (EIS) results and (**f**) Cycle stability of aY20, aY45, and aY165.

Figure 7(a,b) display typical cyclic voltammetry current-voltage (CV) curves and charge-discharge profiles respectively for Y45 sample as an example. The rest of the CV and charge-discharge curves can be found in Supporting Information. The CV curves show symmetrical rectangular shapes and the charge-discharge profiles show nearly symmetric triangular shapes for all scan rates and current densities. This represents good to excellent capacitive performances. Capacitances generally follow the same trend as surface area and pore volume based on the governing formula for capacitors, C = ε·A/d, where C is the capacitance of the supercapacitor, ε is the product of electrolyte dielectric constant and permittivity of free space, A is the surface area between the electrode and electrolyte, and d is the separation distances between ions in the electrolyte and the electrons in the electrodes. As a result, the amount of charge that can be stored, i.e. capacitance, increases with increasing accessible surface^44^. Therefore, aY20 and aY45 with the highest surface areas of all samples give the highest capacitance of up to 113 F g^−1^. The direct correlation between surface area and capacitance can be observed in Fig. 7(c,d), where a summary of the capacitance values is shown. The notable exception to the trend is N165 and Y165 samples. Y165 has a higher surface area when compared to N165 but not its capacitance. Although many factors can cause this discrepancy, at least one of the major factors is the pore characteristics of the two samples. From the gas adsorption-desorption experiments, it has been shown that N samples have larger pores than the Y samples. The Quenched Solid-State Functional Theory (QSDFT) model showed that Y165 has 56% of its pores smaller than 0.64 nm, which was the lower limit of our pore size measurements. N165, however, only has 46% of such small pores. Although solvated potassium ion has a size of 0.31 nm and solvated hydroxyl ion has a size of 0.35 nm^50^ which are much smaller than our 0.64 nm limit, we may still speculate that the larger pores in N165, partly compensated its low surface area for capacitance. Many micropores of Y165 could have blocked electrolyte ions from reaching the carbon electrode surface, reducing the effective surface area on the electrode for capacitance applications^51,52^. Mesoporosity on the other hand, could make the diffusion of electrolyte ions onto carbon electrode surfaces easier, contributing to high capacitance, especially when fast diffusion of ions are required, as in the case with a high scan rate^50,53^. As a result, N165 and Y165 have similar capacitances at a slow scan rate, but as the scan rate increases, N165 gradually outperforms Y165. Similarly, other Y samples generally have poorer rate handling capability in comparison to the N samples. Noticeably, aY165 rate handling capability was the worst followed by aY45. Similar to Y165, the first suspect for explaining the poor rate handling was the kinetic limitation from pore size distribution. However, the DFT results of aY165 and aY45 did not differ much from aY20 which the only other activated sample but with good rate handling capability.

The activated samples were further analyzed using electrochemical impedance spectroscopy. Figure 7(d) shows the Nyquist plots exhibit almost vertical lines at the low frequency region, representing behaviors closer to an ideal capacitor^17,54^. A shallower slope closer to 45° as seen at the mid to low frequency range represents Warburg resistance which indicates the slow diffusion of ions on the surface of the electrodes^55^. One can also find a good indication of the equivalent series resistance when extrapolating the vertical portion of the curve to the x-axis on the Nyquist plot^56,57^. Notably, aY165 curve is shifted to the right relative to aY45 and with aY20 being the furthest left curve. This indicates impedance of aY165 is higher than aY45 then closely followed by aY20. This explains the trend observed with a steeper drop in capacitance vs. scan rate curve in Fig. 7c for aY165 than aY45 and aY20 with the shallowest drop discussed earlier. We believe the main reason in their conductivity differences are due to the different amounts of metal species in the activated samples and thus their capacitance rate handling capability. Indeed, earlier ICP-OES results in Table S1 showed that aY45 and aY165 have the highest amount of metal species from the iron catalyst and the KOH activation process. Specifically, aY165 contains 0.897% iron and 0.535% potassium, the highest, followed by aY45, then aY20 with the least with 0.313% iron and 0.211% potassium.

Because of the promising capacitances, 5000 long term cycling stability was evaluated for all activated samples as shown in Fig. 6(e). Capacitance retentions of 98.3%, 97.2% and 99.8% for aY20, aY45, and aY165 respectively were measured, revealing high cycle stability performances during practical applications.

So far, we have shown supercapacitor properties of samples made from sugar derived carbonaceous materials. To correlate the functionalities of sugar derived activated carbon with real world waste management, the hollow carbon spheres made from woodchip pretreatment liquid effluence was activated and characterized. A surface area of 872 m^2^/g and a pore volume of 0.511 cc/g were obtained. When made into supercapacitor electrodes, capacitance of 109 F/g was measured with a scan rate of 5 mV/s which maintained a 90.5% capacitance retention after 5000 cycles (Figure S4). The desirable result and nearly ideal capacitive behavior reinforced the potential for biorefineries to utilize its biomass waste using simple emulsion-based hydrothermal synthesis for EDL supercapacitor electrode applications as a value-added product.

## Conclusions

We have analyzed the evolution of spherical carbon particles and pore formation mechanisms from sugars via hydrothermal synthesis. The morphologies of these products can be controlled by modifying the composition of the media and thus altering the carbonization mechanisms. Both computational and experimental results show an intriguing effect that carbon morphologies evolve from a poorly defined charcoal material to perfectly shaped spherical carbon as HTC time increases. The size of the spheres can also be controlled by the HTC duration. Because of the differences in reaction mechanisms, surfactant loaded precursor in the emulsion medium self-assembled into hollow spheres (Y sample) whereas in the absence of surfactant in simple hydrothermal reaction medium, the precursors only yield solid spheres (N Sample). In terms of porosity, Y samples have higher surface area and microporosity due to their hollow nature and the layered templating effect caused by the surfactant molecules. In contrast, N samples are both microporous and mesoporous mainly due to the evolution and activation of volatiles during carbonization. The carbon surface area analysis, molecular dynamics simulations, and the measured small-angle x-ray scattering data reveal the templating effect of surfactants on the emulsion-based hydrothermal synthesis of carbons. When these carbons were made into EDL electrodes of supercapacitors, the observed supercapacitances correlated very well with the measured surface areas and exhibited excellent capacitive behaviors. Particularly, the Y samples synthesized for short duration (20–45 minutes) show very high capacity (*ca*. 50–80 F/g) even at high scan rates. The best performing Y samples were then activated with KOH, and surface areas and capacitances further improved up to 1495 m^2^ g^−1^ and 113 F g^−1^, respectively, with a 98.3% capacitance retention for aY20 after 5000 cycles. Finally, we produced supercapacitor electrodes from liquid effluence of steam pretreated woodchips that are typically a byproduct of biorefineries using our reaction pathway. Perfectly hollow spheres can be synthesized from the woodchips. The resulting activated hollow carbon spheres exhibited a desirable surface area of 872 m^2^/g and capacitance of up to 109 F/g. Almost ideal capacitive behaviors were observed with 90.5% capacitance retention after 5000 cycles. Thus, we provide a potential solution for deriving energy harvesting materials from a renewable resource. While the investigation was performed at a laboratory scale, the simplicity of the overall synthesis technique can easily be scaled up to industrial standards. The advantage of this method is threefold: (1) the synthesis technique is simple, (2) the method can be used in parallel with biorefinery unit operation, and (3) the cost of biorefineries will eventually decrease since a waste product can be converted into an energy storage material. We believe this work will influence a change in the practices of biorefineries towards achieving their goal of competing with fossil fuels.

## Methodology

### Hydrothermal synthesis and carbonization

HTC of carbon was conducted from 120 ml 0.5 M sucrose (Diamond Crystal, Savannah, GA) and 0.5 M FeCl_3_ solution. In another case, 0.5 M sucrose and 0.5 M FeCl_3_ solution were made from an emulsion of ultrasonicating 1.2 g sodium dodecyl sulfate (SDS), 24 ml paraffin oil (Merck-KGaA, Darmstadt, Germany), and 96 ml DI water. The hydrothermal reactions were carried out for 20, 45, or 165 minutes. Additionally, liquid effluence of steam pretreated woodchips (obtained from carpentry waste from eastern Tennessee mixed hardwood biomass) at 180 °C for 12 hours was made into an emulsion just like the previous case with SDS and paraffin oil then subsequently HTC for 165 minutes with 1.2 g FeCl_3_. Amount of carbohydrate resulted in the liquid effluence was estimated at around 2.5 g. All chemicals were purchased from Sigma-Aldrich unless noted otherwise. Synthesis was done in a 200 mL PPL lined stainless steel autoclave (Columbia International). The autoclave was placed in an oven at 200 °C for a specific synthesis time. The hydrothermal synthesized solid product was then placed in a quartz tube inside a tube furnace and carbonized at 1000 °C for 20 minutes in a nitrogen atmosphere. The carbonized material was then washed with 0.5 M HCl and water.

### Activation

KOH was ground with the dried samples in a 2:1 (KOH:sample) ratio. Ground samples were then ramped in a tube furnace under a nitrogen atmosphere at the rate of 8 °C per minute to 800 °C and held for 30 minutes. The activated samples were then cooled, washed with water, and dried. The activated ID carbon materials were designated as “aID” carbon (i.e. activated Y20 is named as aY20).

### Characterization

Scanning electron microscope (SEM) images were collected with a Hitachi S4800. Carbon sphere diameters were measured by Image J software and characterized by the mean and standard deviation of 10 randomly chosen spheres within a sample. Transmission electron microscope (TEM) images were taken using a Zeiss Libra 120 Transmission Electron Microscope operating at 100 kV. Samples were dispersed onto a carbon film coated copper grid before analysis. Thermal gravimetric analysis was investigated by a Q500, TA instruments. For each sample, *ca*. 15 mg was used for measurement onto a platinum pan. Temperature was ramped to 105 °C at 10 °C/min, held for 30 min, and ramped to 1000 °C at 7.5 °C/min in nitrogen atmosphere. Inductively coupled plasma optical emission spectrometry was outsourced to Galbraith Laboratories Inc., a commercial analytical chemistry laboratory service in Knoxville, TN. Nitrogen adsorption desorption experiments were carried out with a Quantachrome Autosorb iQ at 77 K. Surface area, pore size distribution, and pore volume were determined using the Quenched Solid Density Functional Theory (QSDFT)^58^. For capacitance measurements, the carbonaceous material was first mixed with a conductive carbon black (Timcal super C45) at 8:1 ratio then with 10 wt. % aqueous Polytetrafluoroethylene (60% dispersion in water). The mixture was then mixed in ethanol to form a paste. The paste was then coated on two 7/16^th^ in. diameter Ni-foam circles and dried overnight. These current collectors were then pressed and used as electrodes in symmetrical two-electrode cells with 6 M KOH as electrolyte. Filter paper served as the separator. Two stainless steel rods were used to clamp on the electrode-filter paper-electrode complex and the complex was housed inside a Teflon Swagelok cell. VersaSTAT 4 (Princeton Applied Research) was used to perform cyclic voltammetry, charge-discharge, and electrochemical impedance spectroscopy (EIS) experiments. A 0 to 0.8 V voltage window was used. Scan rates varied from 10 mV s^−1^ to 200 m V s^−1^ and from 200 mA g^−1^ to 2000 mA g^−1^. EIS was conducted at a frequency of 500 kHz to 50 mHz with an amplitude of 10 mV. The specific capacitances were calculated from C = 2q/mE, where E is the voltage window of 0.8 V, m is the mass of the carbon sample used, and q is the charge accumulated calculated from VersaStudio software. Cycle stability was conducted on a Arbin battery cycler (Arbin Instrument) at 500 mA g^−1^.

Small-Angle X-ray Scattering (SAXS) data were acquired at the Center for Nanophase Materials Sciences (CNMS) in Oak Ridge National Laboratory on an Anton Paar SAXSess mc^2^. The scattered beam was recorded on a CCD detector (PI-SCX, Roper) with a pixel resolution of 2084 × 2084 and pixel dimensions of 24 × 24 µm^2^. The data collection time was 20 minutes. For the measurements, the X-ray was generated at 40 kV/50 mA at a beam wavelength of *λ* = 1.541 Å (Cu Kα radiation). The generated X-ray beam was slit-collimated using a Kratky camera giving rise to the beam size of 18 mm (Length) × 0.4 mm (Width) and the collected SAXS data were desmeared and expressed as intensity versus *Q*, where *Q* = (4*π* sin*θ*)/*λ* after subtraction of detector dark current and background scattering.

### Computational Model

Coarse-grained molecular dynamics (CGMD) simulations were performed on a dilute mixture of sugar and SDS surfactant. The sugar molecules were modeled as short polymer chains of 15 monomeric units with 4 hydrophilic monomers with charges on the polymer backbone. The charges on the polymer chains allow the chain to be slightly polar thereby mimicking the polar hydroxyl groups of the sugar molecules. The charge sites are converted to neutral once aggregates are formed to mimic the fully hydrophobic carbonaceous sugar molecules after HTC. The SDS surfactants were modeled as 12-mer polymer chains with 1-mer hydrophilic polar head and 11-mer hydrophobic tails has been done in previous CGMD studies^59,60^. While the experiments were performed in both water and emulsion (in SDS), we performed only one set of simulation in SDS (emulsion in experiments). The purpose of the simulation was to understand the self-assembly of carbonaceous bead formation irrespective of the presence of absence of SDS, we chose the latter. The interactions between the neutral monomers were modeled using Lennard-Jones (LJ) force-field (FF) while the charge interactions were modeled using explicit Coulomb interactions. Each monomer bead was represented by mass, *m*, and Lennard-Jones bead diameter, *σ*. For the simulations, we considered the mass and LJ diameter equal to 1 and 0.97 respectively, same for all monomers. As the variation of *m* and *σ* of sugar and surfactant monomers are relatively small, hence this choice *m* and *σ* would provide critical self-assembly information without drastically altering the fundamental physics. The model system consisted of 2000 polymer chains and 2000 surfactant molecules in a periodic box of 100*σ* × 100*σ* × 100*σ* at a density, 0.064/*σ*^3^. In experiments, the hydrothermal carbonization process strips off the charges on the sugar and SDS molecules. Therefore, the most realistic way to computationally model hydrothermal carbonization would be to strip off the charges after a certain simulation time. Hence, we modeled the carbonization process by stripping off the charges from both the polymers and surfactants after equilibrating for 5 million LJ time-steps. By deleting all the charges from the system, the interaction between the monomers becomes solely hydrophobic, representing a purely carbonaceous material. The polymer chains and surfactant molecules undergo self-assembly during equilibration, however, to achieve equilibration for a fully hydrophobic (no-charge scenario) system, we ran for 3 million more time steps. All the simulation parameters are in reduced units. The temperature is fixed at, T* = $1.0{k}_{B}T/\varepsilon$ and the simulation time step is fixed at, Δt = 0.01*σ*. The visualization of MD trajectories were generated by VMD code^61^ and the structural analysis was performed using our in-house code.

## Electronic supplementary material

Supplementary Information
