# Supplementary material for: Amending the Structure of Renewable Carbon from Biorefinery Waste-Streams for Energy Storage Applications
Source: Sci Rep. 2018 May 29;8:8355. doi: 10.1038/s41598-018-25880-0 (PMC5974299; doi:10.1038/s41598-018-25880-0)
Supplement: Supplementary file 1 — Supplementary Information [file 41598_2018_25880_MOESM1_ESM.docx]

**Supplementary Information**

**Amending the Structure of Renewable Carbon from Biorefinery Waste-Streams for Energy Storage Applications**

Hoi Chun Ho,^1,2^ Monojoy Goswami,^3,4^ Jihua Chen,^3^ Jong K. Keum,^3,5^ and Amit K Naskar^1,2^*

*^1^Carbon and Composite Group, Materials Science and Technology Division, Oak Ridge National Laboratory, Oak Ridge, TN. 37831 USA.*

*^2^* *The Bredesen Center for Interdisciplinary Research and Graduate Education, The University of Tennessee, Knoxville, TN. 37996 USA.*

*^3^Center for Nanophase Materials Sciences, Oak Ridge National Laboratory, Oak Ridge, TN. 37831 USA.*

*^4^Computer Science and Engineering Division, Oak Ridge National Laboratory, Oak Ridge, TN. 37831 USA.*

*^5^Neutron Scattering Division, Oak Ridge National Laboratory, Oak Ridge, TN. 37831 USA*.

Correspondence to: [naskarak@ornl.gov](mailto:naskarak@ornl.gov)

**Table S1.** Iron and potassium contents of all samples analyzed by Inductively coupled plasma optical emission spectrometry (ICP-OES). The minimal redox peaks in the cyclic voltammetry experiments and the low metal contents lead us to believe pseudocapacitance does not play a major role in our results. With that said, metal species did affect electrodes’ conductivity confirmed by Electrochemical impedance spectroscopy and 5000 cycle stability results.


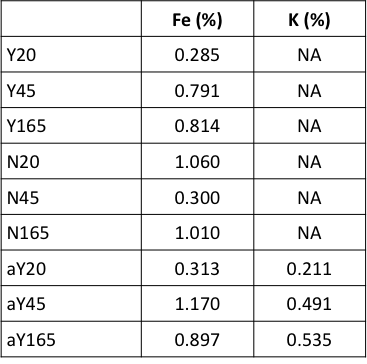


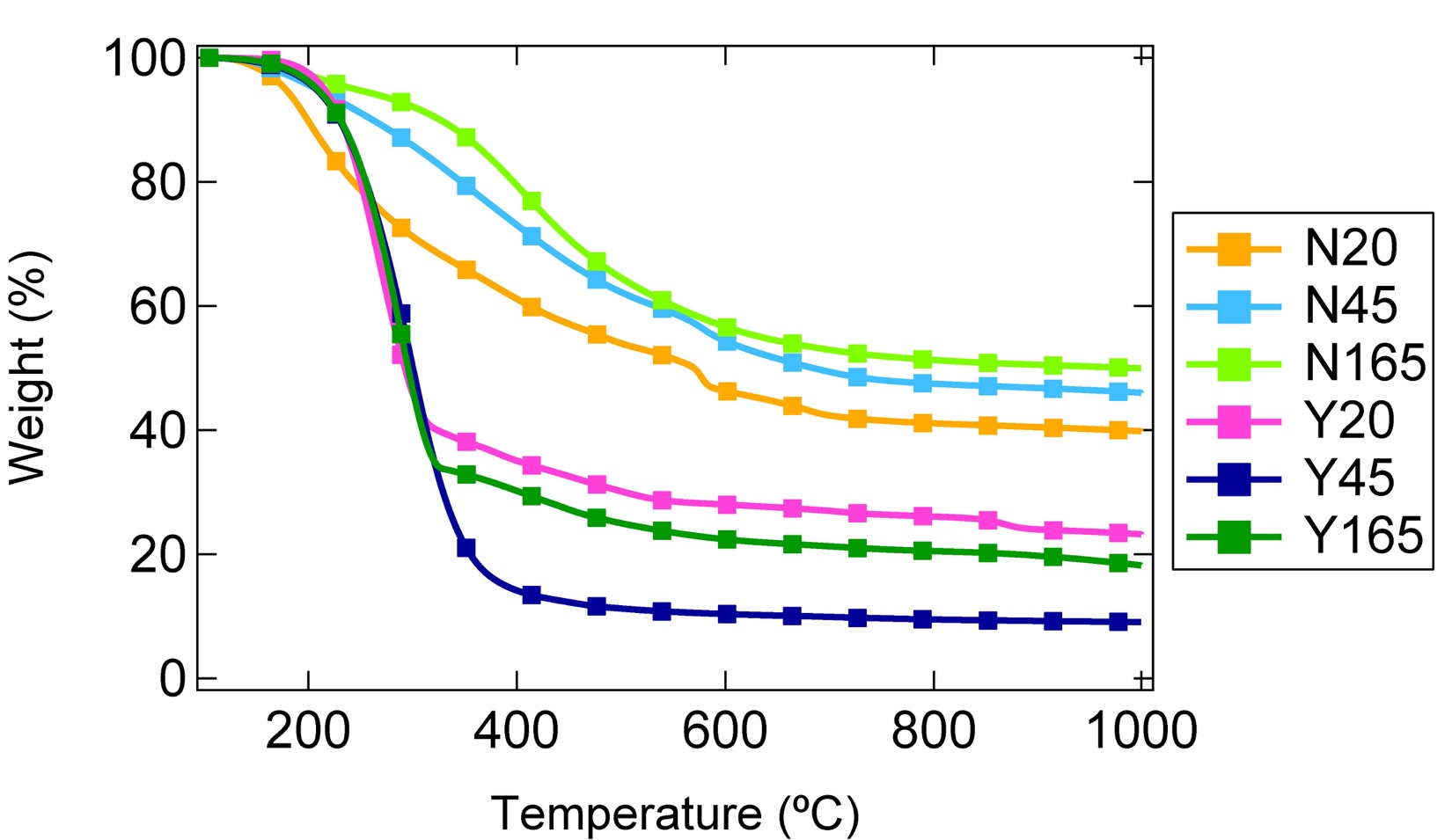


**Figure S1.** Thermogravimetric analysis of all hydrothermal synthesized samples prior to high temperature carbonization. Onset of thermal decomposition, thermal stability, and yield all follow the trend with 165>45>20 for N samples due to longer hydrothermal synthesis duration provides larger degrees of stabilization. The same conclusion however, cannot be drawn for the Y samples mainly because of the residue paraffin oil being burned off at ca. 300°C. All samples saw decomposition and carbonization up till *ca.* 700°C. Weights stay largely constant after *ca.* 700°C. N samples have noticeably steeper slopes and weight loss at *ca.* 400°C to 700°C due to volatile evolution which are less pronounce in the Y samples due to different carbonization mechanism.

**Figure S2.** Electrochemical testing of the carbonaceous products when used as supercapacitor electrodes. Cyclic voltammetry of (a) Y20, (b) Y165, (c) N20, (d) N45, (e) N165, (f) aY20, (g) aY45, and (h) aY165 at 10, 20, 50, 100, and 200 mV s^-1^ scan rates.

c)

e)

a)

b)

f)

g)

**Figure S3.** Electrochemical testing of the carbonaceous products when used as supercapacitor electrodes. Charge discharge curves of (a) Y20, (b) Y165, (c) N20, (d) N45, (e) N165, (f) aY20, (g) aY45, and (h) aY165 at 200, 500, 1000, and 2000 mA g^-1^ current densities.

**Figure S4.** Surface characterization and electrochemical testing of woodchip pretreatment liquid effluence-derived activated carbon. (a) Isotherm, (b) Pore size distribution, and density functional theory calculation reveals an abundance in microporosity (72%) and a smaller amount of mesoporosity (28%). (c) Cyclic voltammetry with symmetric rectangular shapes (at 10, 20, 50, 100, and 200 mV s^-1^ scan rates), (d) Charge discharge profiles with symmetric triangular shapes (at 200, 500, 1000, and 2000 mA g^-1^ current densities), and (e) Electrochemical impedance spectroscopy Nyquist plot indicating almost ideal capacitive behavior. (f) Capacitance vs. scan rates. (g) 5000 cycle stability with 90.5% capacitance retention supporting electrode’s practical application.
